# Gene ontology analysis of pairwise genetic associations in two genome-wide studies of sporadic ALS

**DOI:** 10.1186/1756-0381-5-9

**Published:** 2012-07-28

**Authors:** Nora Chung Kim, Peter C Andrews, Folkert W Asselbergs, H Robert Frost, Scott M Williams, Brent T Harris, Cynthia Read, Kathleen D Askland, Jason H Moore

**Affiliations:** 1 Institute for Quantitative Biomedical Sciences, Department of Genetics, Dartmouth Medical School, One Medical Center Dr., Lebanon, NH 03756, USA; 2 Department of Psychiatry and Human Behavior, Butler Hospital, Brown University, 345 Blackstone Blvd, Providence, RI 02906, USA; 3 Department of Cardiology, Division Heart & Lungs, University Medical Center Utrecht, Utrecht, the Netherlands; 4 Department of Neurology, 4000 Reservoir Rd, Georgetown University Medical Center, Washington, DC 20057, USA

## Abstract

**Background:**

It is increasingly clear that common human diseases have a complex genetic architecture characterized by both additive and nonadditive genetic effects. The goal of the present study was to determine whether patterns of both additive and nonadditive genetic associations aggregate in specific functional groups as defined by the Gene Ontology (GO).

**Results:**

We first estimated all pairwise additive and nonadditive genetic effects using the multifactor dimensionality reduction (MDR) method that makes few assumptions about the underlying genetic model. Statistical significance was evaluated using permutation testing in two genome-wide association studies of ALS. The detection data consisted of 276 subjects with ALS and 271 healthy controls while the replication data consisted of 221 subjects with ALS and 211 healthy controls. Both studies included genotypes from approximately 550,000 single-nucleotide polymorphisms (SNPs). Each SNP was mapped to a gene if it was within 500 kb of the start or end. Each SNP was assigned a p-value based on its strongest joint effect with the other SNPs. We then used the Exploratory Visual Analysis (EVA) method and software to assign a p-value to each gene based on the overabundance of significant SNPs at the α = 0.05 level in the gene. We also used EVA to assign p-values to each GO group based on the overabundance of significant genes at the α = 0.05 level. A GO category was determined to replicate if that category was significant at the α = 0.05 level in both studies. We found two GO categories that replicated in both studies. The first, ‘Regulation of Cellular Component Organization and Biogenesis’, a GO Biological Process, had p-values of 0.010 and 0.014 in the detection and replication studies, respectively. The second, ‘Actin Cytoskeleton’, a GO Cellular Component, had p-values of 0.040 and 0.046 in the detection and replication studies, respectively.

**Conclusions:**

Pathway analysis of pairwise genetic associations in two GWAS of sporadic ALS revealed a set of genes involved in cellular component organization and actin cytoskeleton, more specifically, that were not reported by prior GWAS. However, prior biological studies have implicated actin cytoskeleton in ALS and other motor neuron diseases. This study supports the idea that pathway-level analysis of GWAS data may discover important associations not revealed using conventional one-SNP-at-a-time approaches.

## Findings

Amyotrophic Lateral Sclerosis (ALS) is a neurological disease that causes motor neuron degeneration, leading to paralysis and eventually death. Around 5,600 people are diagnosed with ALS each year with the incidence rate of two per 100,000 a year [1]. Despite the relatively low incidence rate compared to more prevalent diseases such as Alzheimer’s and Parkinson’s, ALS is a devastating disease with the average life expectancy of only two to five years from the time of diagnosis. Unfortunately, genome-wide association studies (GWAS) across multiple cohorts have not revealed replicable, genome-wide significant single-nucleotide polymorphisms (SNP) associations that could provide additional clues about the etiology of ALS [2-7]. It is important to note that the prior studies assumed a simple genetic architecture with an analytical approach that was designed to only detect single SNPs with large effects independent of the genomic background or the ecological context of the subjects being studies. It is our working hypothesis that the genetic architecture of sporadic ALS is complex and likely to be influenced by gene-gene interactions [8]. We further hypothesize that patterns of gene-gene interactions influence ALS susceptibility at the pathway level with individual gene effects playing a smaller role. The goal of the present study was to determine whether patterns of pairwise genetic effects aggregate in specific functional groups as defined by the Gene Ontology (GO). We briefly outline here our bioinformatics approach and then summarize the findings.

**Figure 1:**
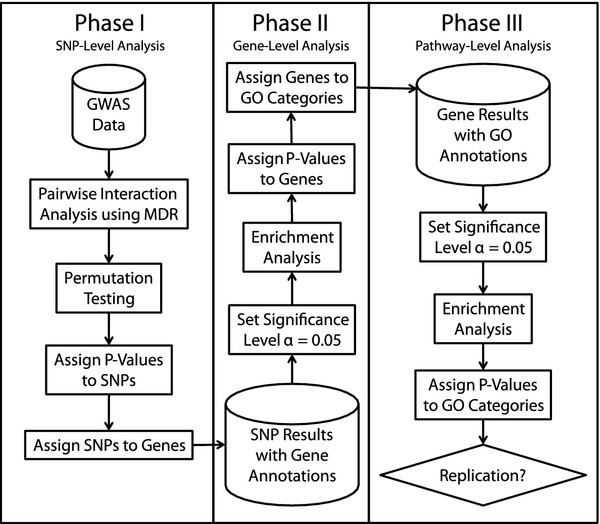
Flowchart summarizing the three phases of our Gene Ontology analysis strategy.

Figure 1 provides a flowchart for our bioinformatics analysis. The analyses are split into three phases. The first phase is a SNP-level analysis that consists of data processing and gene-gene interaction analysis. The second phase is a gene-level analysis that determines whether each gene has more statistically significant SNPs than expected by chance. The third phase is a pathway-level analysis that determines whether each GO category has more statistically significant genes than expected by chance. This is followed by an assessment of replication between the independent GWAS studies of sporadic ALS. We briefly summarize the approach for each of these phases.

### Phase I: SNP-level analysis

We ascertained and processed each GWAS dataset for sporadic ALS as described previously by Greene et al. [8]. The detection data consisted of 276 subjects with ALS and 271 healthy controls [3] while the replication data consisted of 221 subjects with ALS and 211 healthy controls [4]. Both studies included genotypes from approximately 550,000 single-nucleotide polymorphisms (SNPs). We then carried out a Multifactor Dimensionality Reduction (MDR) analysis to estimate the pairwise additive and nonadditive effects of SNPs on ALS susceptibility in each study. MDR is a machine learning method that was designed specifically to detect and characterize gene-gene interactions using constructive induction [9-12]. The MDR approach combines multiple SNPs at a time and then estimates the accuracy of a naïve Bayes classifier for predicting case–control status. The advantage of this approach is that it doesn’t assume a particular genetic model and can thus simultaneously detect bot additive and nonadditive effects. Approximately 151 billion pairwise genetic effects were modeled in each data set.

Statistical significance of the accuracy of MDR models was evaluated using permutation testing. The goal of the permutation test was to estimate the distribution of MDR model accuracies under the null hypothesis of no association. Here, we randomized case–control labels 1000 times to create 1000 data sets consistent with the null hypothesis. In each null data set we estimated accuracies for all pairwise MDR models and selected the best one. Using this null distribution we derived the critical value of the accuracy statistic at an α = 0.05 significance level. Permutation testing revealed a critical value of the MDR classification accuracy of 0.629 for the detection data and 0.640 for the replication data at a genome-wide α = 0.05 significance level. This is a genome-wide significance level because it was derived from considering all possible pairs of SNPs with MDR on each null data set. Thus, the same numbers of models considered in the real data were also considered in each null data set. P-values were derived from the two null distributions for every MDR model in the detection and replication data sets.

The next step was to map the 151 billion pairwise MDR model p-values to each of the approximately 550,000 individual SNPs. We chose the best MDR model for each SNP and assigned that SNP the corresponding MDR model p-value. We then assigned SNPs to genes if they were within 500 kb upstream of a gene start or 500 kb downstream of a gene end. The rationale for this window size was to ensure inclusion of regulatory SNPs [13] that are expected to play an important role in gene-gene interactions due to effects of DNA sequence variation on transcriptional networks [14].

### Phase II: Gene-level analysis

The gene-level enrichment analysis was performed using the Exploratory Visual Analysis (EVA) methodology and software. EVA was designed specifically for the visualization and analysis of statistical results from gene expression studies [15]. We have more recently expanded EVA for the analysis of SNP data [16,17]. The goal of phase II was to determine whether a gene has an overabundance of statistically significant SNPs. Here, we used EVA to assess whether SNPs with genome-wide significant MDR p-values of 0.05 or less are overrepresented given the size of the gene. Statistical significance is determined in EVA using a Fisher’s exact test based on the hypergeometric distribution. P-values are then assigned to genes and genes assigned to GO categories. For the GO assignment, we used the annotations from the Molecular Signatures Database or MSigDB, version 3.0 [18].

It is important to note that a limitation of this approach is that it will likely miss pathways (GO categories) that are comprised mostly of genes that each has only one or a few significant SNPs. Our gene-level analysis makes the assumption that genes likely to show pathway-level effects (see Phase III below) are likely to be involved in multiple pairwise genetic associations within the same gene region and/or with other genes in the same pathway. Within a gene region, multiple SNPs in a promoter or enhancer sequence might synergistically influence the affinity of a protein to bind to DNA. It is also possible that one SNP influencing protein binding in a promoter might synergistically interact with another influencing protein binding at an enhancer through protein-protein interactions and chromatin looping. Between two genes in different regions, synergy among SNPs could arise as the result of amino acid changes that change physical interactions of their corresponding protein products in a regulatory region of yet a third gene. Many more examples of regulatory sequence interactions are possible. We have previously speculated about such complex regulatory interactions [14].

### Phase III: Pathway-level analysis

The pathway-level GO enrichment analysis was also performed using the EVA methodology and software as described above. Here, we used EVA to assess whether genes with p-values of 0.05 or less are overrepresented in a given pathway accounting for its size. A GO category was considered statistically significant at an α = 0.05 level. Gene Ontology categories that had p-values less than or equal to 0.05 in both the detection and replication datasets were considered replicated and carried forward for presentation and discussion. We explicitly used replication to control for false-positives due to multiple testing across GO categories.

### Results

The bioinformatics analysis strategy described above and in Figure 1 revealed two replicated and statistically significant GO categories. The first, ‘Regulation of Cellular Component Organization and Biogenesis’, a GO Biological Process, had p-values of 0.010 and 0.014 in the detection and replication studies, respectively. The second, ‘Actin Cytoskeleton’, a GO Cellular Component, had p-values of 0.040 and 0.046 in the detection and replication studies, respectively. We focus here on interpretation of the role of actin cytoskeleton in sporadic ALS because it is a subcomponent of the set of cellular component organization and biogenesis genes.

### Discussion

Actin is a highly conserved protein that forms microtubules in cells. Actin is an important part of the cytoskeleton and participates in or makes possible a number of cellular functions including protein trafficking and cell motility. The biological role of actin cytoskeleton in motor neuron diseases has long been recognized [19]. A recent review by Julien et al. [20] provides an overview of the many studies that have implicated cytoskeletal defects in ALS. Interestingly, SNPs in actin cytoskeleton genes have not replicated across the published GWAS for sporadic ALS [2-7]. They have, however, been implicated in other diseases such as multiple sclerosis [21]. Given the known biological basis for actin cytoskeleton in motor neuron diseases, including ALS, and the results presented here, we conclude that genes in this GO category should be examined more carefully in GWAS for sporadic ALS. In particular, such a careful examination should entail an analytical approach that is robust to the detection of pathway replication in the absence of gene replication. The results of this study support the idea that a bioinformatics approach to GWAS analysis that embraces the complexity of the genotype-phenotype map has the potential to reveal interesting new associations that have not been discovered using one-SNP-at-a-time approaches.

## Availability

The EVA and MDR software packages are freely available from the authors. More information can be found at http://www.epistasis.org.

## Competing interests

The authors declare they have no competing interests.

## Authors’ contributions

NCK assisted with method development, carried out the analyses, interpreted the results and drafted the manuscript. PCA assisted with method development and with the programming necessary to implement the method. FWA, HRF, SMW, CR and KDA assisted with method development, interpreted the results and drafted the manuscript. BTH provided the data, interpreted the results and drafted the manuscript. JHM assisted with method development, assisted with the analyses, interpreted the results and drafted the manuscript.
